# Lesbian shared IVF: the ROPA method: a systematic review

**DOI:** 10.1097/j.pbj.0000000000000202

**Published:** 2023-04-10

**Authors:** Pedro Brandão, Nathan Ceschin

**Affiliations:** a Department of Reproductive Medicine, Instituto Valenciano de Infertilidad, Valencia, Spain; b Faculdade de Medicina da Universidade do Porto, Porto, Portugal; c Feliccita Fertility Clinic, Curitiba, Paraná, Brazil

**Keywords:** fertilization in vitro, Lesbian, ROPA, Shared IVF

## Abstract

The ROPA (Reception of Oocytes from PArtner) method, also known as lesbian shared IVF (in vitro fertilization), is an assisted reproduction technique for female couples, in which one of the women provides the oocytes (genetic mother) and the other receives the embryo and gestates (gestational mother). As a double parented method, it is the only way lesbian women may biologically share motherhood. This is a narrative review of data concerning ROPA published in PubMed, Scopus, and Cochrane Library. A total of 35 articles were included, 10 about motivations for undergoing ROPA, 13 about ethics or legislation, 4 about motherhood, and 8 studies reporting clinical outcomes. Despite being used for more than a decade, there is a paucity of data regarding this technique in scientific literature. Most women choose this technique to share biological motherhood, but medical issues may also justify its use. Many ethical and legal issues are still to be solved. Despite the small number of studies, data regarding the outcomes of this technique and the resulting motherhood are reassuring.

## Introduction

The ROPA method (Reception of Oocytes from PArtner), also known as lesbian shared IVF (in vitro fertilization), consists of an assisted reproduction technique (ART) for female couples, in which one partner provides the oocytes (donor, egg provider, giving partner, or genetic mother) and the other receives the embryo and gestates (recipient, receiving/gestating partner, or gestational mother).^1^ It may also be called lesbian shared IVF, intrapartner oocyte donation, lesbian reciprocal IVF, or partner IVF.^2^

As for artificial insemination (AI) or IVF with donated semen, both women will be legal mothers, but from a biological point of view, they are single parented methods.^3-5^ On the other hand, ROPA is a double parented method because it allows both women to take an active role in the conception of the newborn.^6^

In practical terms, it is an IVF with donated semen but in which the embryo is transferred to the uterus of the partner. It not only allows couples to share biological motherhood but it may also be used in case one partner has impaired ovarian function and the other has any condition precluding gestation. In addition, it is useful in cases of transgender patients who underwent gender physical reassignment after fertility preservation.^7^

The process of the ROPA method is quite similar to conventional IVF. However, in this case, the embryo will not share any biological links with the gestational carrier. This is similar to IVF treatments with donated oocytes, but in the latter, donors are usually selected young women. In addition, either being accepted as a donation of oocytes or embryos, this process is entirely made within a couple.

Another interesting point of this treatment is its range of possibilities because couples may opt to do it unidirectionally, which means one of them will be donor and the other will be recipient, but patients may also play both roles, either at the same time (reciprocal ROPA) or in different occasions (reverse ROPA). In addition, patients may opt to invert roles after an unsuccessful cycle to try to improve the outcomes.

Although the clinical and laboratory aspects of this technique are quite similar to conventional IVF, little has been described about the subject. There are also important ethical and legal aspects related to this technique. There is still little information in the literature about its ethical aspects. In addition, many legal aspects of assisted reproduction for gay couples are not contemplated in national legislation, reason why the daily practice is often based on assumptions derived from the law for heterosexual couples.^8^ The same goes for aspects related to lesbian motherhood, as much of the current evidence is based on adoption or single parented methods.^9^

## Objective

The aim of this work was to review published data about the ROPA method, in a holistic approach, including all the studies concerning the various aspects of this technique.

## Methods

A review of all articles listed in PubMed, Scopus, and Cochrane Library was conducted in November 2022 using the query: ropa or “shared motherhood” or “reciprocal ivf” or “reciprocal in vitro fertilization” or (lesbian or lesbians or gay or lgbt or homosexual or “same sex” or “same-sex”) and (ivf or “in vitro fertilization” or “in vitro fertilization” or “assisted reproduction” or “assisted reproductive”).

Editorials, letters to the editor, comments, corrigenda, replies, book chapters, and study protocols were excluded. The remaining works were included despite the type or methodology of research. Articles written in English, Portuguese, Spanish, or French were included. No limit of date was set. References of the selected articles were thoroughly reviewed to include other potentially related articles.

## Results

### Study appraisal

As a result of the search, 1415 works were retrieved (PubMed: 558, Scopus: 831, Cochrane Library: 26). Duplicates were removed (n=382). All articles' titles and/or abstracts were analyzed. Studies not related to the study question (n= 710) were excluded. From the remaining articles, the following were excluded for being related to LGBT reproduction but not mentioning the ROPA method: 83 were related to lesbian reproduction in general, 19 referred to male couples' reproduction, 69 were about transgenders, and 109 were about the LGBTQ+ (lesbian, gay, bisexual, transexual, queer, and more) community reproduction in general. Nine other works were not included for being reviews, letters to the editor, comments, corrigenda, or book chapters. Two additional articles were excluded due to language issues (1 in Croatian and 1 in German). The references of the works retrieved were checked, and 3 additional articles were added, making a total number of 35 articles included.

**Figure 1.**
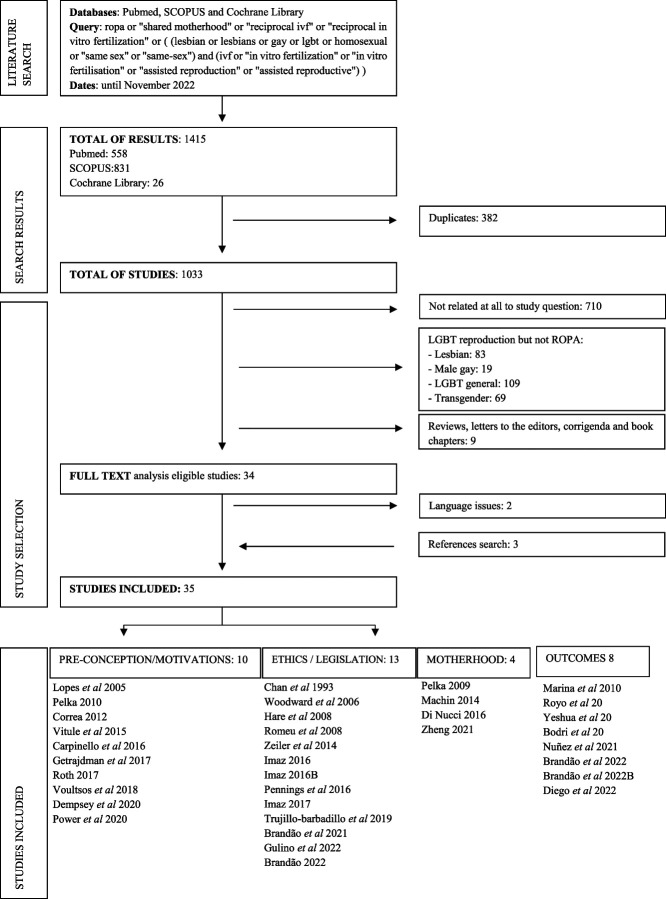
The search process flowchart.

The included articles were divided in 4 groups according to the main subject of their content: 1—preconception issues and motivations to undergo a ROPA treatment, 2—ethics and legislation, 3—studies about resulting motherhood, and 4—quantitative studies (Figure 1).

### Preconception issues and motivations for choosing ROPA

There is a growing number of lesbian couples worldwide searching for the ROPA method.^2,10^

The main reasons for women to choose single parented methods such as AI or IVF are the cost and simplicity of the treatment (especially the former), lack of availability of more complex treatments (usually due to legal issues), and lack of information.^11-13^ Indeed, studies report that the main reason for lesbian women to quit treatments or not to pursue ART are the costs and the lack of insurance coverage.^14^

There are two main reasons why women may undergo a ROPA treatment—to share biological motherhood and for medical indications.^15,16^

Medical indications may include poor oocyte quality, low ovarian reserve, or a genetic disease of the gestational mother. On the other hand, the genetic mother has uterine disorders or any medical issue contraindicating pregnancy.^11,17^

Nevertheless, in most cases, the reason for choosing this method is to share biological motherhood.^18^ In theory, this could improve the mother-child bond, although studies are quite contradictory in this regard. In addition, the biological connection may facilitate the answer to the question “who is the mother?”—it will always be “both.”^19^ Studies show that patients believe ROPA may strengthen their relationship with their partners. Some patients also say that they chose to undergo this treatment to fulfill their partners' wishes to experience a “true shared motherhood.”^20^

ROPA allows women to choose the role they are playing, avoiding those they do not want.^12,19^For instance, a woman may have a strong desire to have genetic related children but may not want to be pregnant.^20,21^ This method is also a way for both to share the burden of the reproductive treatments—at a physical, mental, and emotional level and not one of them to be a mere spectator (one of them will have to undergo ovarian stimulation and oocyte retrieval, while her partner will carry out pregnancy).^19^ Some patients also say that including both partners in the treatment may help omitting the contribution of the donor sperm and the assistance of the IVF procedures and clinicians.^21^

Interestingly, some women believe this method may paradoxically be a setback in the process of accepting nonbiological motherhood, particularly within the LGBTQ+ community. This could ultimately have discriminatory consequences both for couples who have opted for single parented methods or adoption, as well as for male or trans couples, in which shared motherhood may not be possible.^19^

Some couples have it clear when it comes to decide which ART they are going through or which role to play, while others come to the fertility clinic with no previous conceptions.^22^ There are some reports of a practice called “mixing of eggs,” which consists of randomly mixing the eggs of both women so no one knows whose fertilized eggs are.^22^

One group studied a sample of 242 lesbian couples undergoing ART, addressing their plans priori to fertility workup. A small percentage (11.8%) of couples had the preconception intention to undergo double parented treatment. Interestingly, all these couples intended to do the treatment in a single direction, ie, no couple had the initial intention of doing a reciprocal or reverse ROPA. In the end, only 4.1% of the couples had ROPA treatment. However, in 40% of these couples, both partners became pregnant, by reciprocal, reverse ROPA, or using the embryos of one of them to get both pregnant.^14^

Another study included all the couples treated in a fertility clinic in Spain in a 2-year period time and found similar results. From the 129 couples included, only one-third had no condition potentially affecting fertility, including advanced age. Most couples had initially decided to undergo AI or IVF, and the majority kept their plans, as opposed to 38% of the couples who decided to the ROPA method who changed plans. In the end, 11% of the couples underwent ROPA. They also found that passive patients in one-way reproductive techniques were 2 years older than active patients, but no differences were found within ROPA patients.^23^

### Ethics and legislation

In accordance with the principle of autonomy, it is obvious that the ROPA method promotes the autonomy of the patients who choose to undergo this treatment.^15,24^

Regarding the principle of beneficence, ROPA is a means to do good for women who long to have a child together. In the same way, if we assume that existing is better than not existing, we also can assume that any treatment that leads to the creation of a new being will be of his or her best interest too.^15,25^

In relation to the principle of nonmaleficence, there was some awareness about a child being raised in a lesbian family. Studies consistently show that the development of children within a homosexual family is not worse, being in some respects even better than in heterosexual couples. The risks associated with this treatment are the same as for any IVF treatment and their subsequent pregnancies. In the same way the risks of IVF are generally accepted, there is no reason not to accept the risks of the ROPA method. Furthermore, unlike surrogacy and oocyte donation where the risks of pregnancy and ovarian stimulation respectively are for a third party, with ROPA, the entire burden falls merely on the interested parties.^15^ However, if an older woman, with lower ovarian reserve, wants to be the donor, adequate medical counseling must be given so that the risks and benefits are weighed.^22,25^

Bearing in mind that these treatments are performed with the intention of doing good to both patients and their offspring, the ROPA method can be considered a legitimate medical procedure. Similarly, to respect the principle of justice, in a society that accepts relationships and families regardless of gender or sexual orientation, ROPA should be accepted in the same way as any other assisted reproduction treatment.^25,26^ It is true that in most cases there is a viable alternative, such as AI, with lower success rates but also with lower risks and costs for patients. However, just as heterosexual couples may choose to perform an ICSI with their own semen in a case of severe male factor, when they could simply resort to an AI with donated semen, a female couple may also want to follow a treatment that involves both mothers and their own gametes.^11,19^

Some question whether it is licit to perform a treatment without a medical indication, in case ROPA is used exclusively to share biological motherhood.^27^ Nevertheless, older patients who resort to medically assisted procreation exclusively because of their age, do not have any reproductive dysfunction, but rather the physiological ovarian insufficiency associated with female aging. Given that this is a physiological process, and in the absence of a pathological process, one may assume that there is no medical indication for these treatments as well. The same happens with the elective fertility preservation; these women undergo treatment and its inherent risks without a medical indication, merely for postponing motherhood.^15^ These issues bring an interesting point to reflect on, whether the term “reproductive treatment” is somewhat misleading because assisted reproduction does not treat the underlying disorder, but rather help to get pregnant. Therefore, it is questionable whether a broader term should be adopted.^19^

As the name suggests, some see the ROPA method as an oocyte donation. In fact, the acronym may be erroneous, given that a donation implies giving up something for someone else. This is not the case; none of the mothers abdicate the right to motherhood nor is this right attributed to anyone else. Similarly, there is no third party granting patients this right. Furthermore, in a donation process, the donor could never claim the right to maternity. In addition, ROPA would not be possible in countries where the donation is anonymous and would imply the same requirements regarding genetic screening and age limit.^28^ As a matter of fact, in the end, this method may be a way to avoid a third party donation in cases of poor ovarian reserve.^11,19,29^

ROPA may also be regarded as an embryo donation. The same considerations made to oocyte donation would apply. However, the embryo is already the result of the union of gametes with the complete genetic material of the future human being. As such, the value attributed to the embryo is different from that attributed to oocytes and may have its own ethical and legal implications. In some countries, embryo donation is only possible if they are left over from another reproduction treatment. In this case, ROPA treatments would have to be initiated as conventional IVF cycles which would later be changed to ROPA once the embryos were created.^11^

A third way to regard ROPA treatment is as a surrogacy. However, with ROPA, the recipient is carrying her own child and will not give up her maternity rights.^30^ Nor can it be said that it presents an increased risk associated with pregnancy because whatever treatment route patients choose, one of them will ultimately have to become pregnant and assume these risks anyway. Moreover, the ROPA method allows the couple to choose the patient with the best reproductive prognosis, both at the ovarian and uterine level, which, ultimately, can lead to a reduction in obstetric risks.^11^

Given the different points of view regarding LGBT reproduction, different cultures, and numerous ethical issues related to ROPA, national legislation varies greatly between countries. Spain, for instance, was the third country in the world to liberalize the marriage of same-sex couples and the first to accept adoption by homosexual couples, being therefore a very open country when it comes to gay reproduction. In many countries, such as Portugal or Spain, the law provides that female couples may undergo assisted reproduction, but it does not specifically address ROPA. If so, one may assume that it is not forbidden, which implicitly makes this treatment not illegal in these countries.^31-33^

On the other hand, some countries have specific legislation concerning ROPA and state that the rules of oocyte donation do not apply, given that it is a donation within a couple.^34^ In Spain, initially motherhood was automatically recognized only to the parturient and only after delivery, which implied that the donor mother had to ask for the adoption of the child after birth. However, the law was ratified a posteriori, allowing maternity to be defined (for both) during pregnancy.^33,35^

As of the end of 2021, ROPA could be performed with no restrictions in 13 European countries—Austria, Belgium, Finland, France, Iceland, Ireland, Malta, Netherlands, Norway, Portugal, Spain, Sweden, and the United Kingdom. In Denmark, it was possible to perform a ROPA if there is a medical reason justifying this treatment.^36^

Irrespectively of all the advances and progressive laws, there is still a large part of the world that does not accept the registration of a child in the name of a single person or a homosexual couple. In this legal context, the ROPA method can bring an added difficulty in defining legal maternity because the parturient is neither the genetic mother of the child nor a mother resulting from a third party donation process.^27^ This can become an even greater problem if the couple is not married or in a case of divorce and the issue of child custody is raised.^37^ In addition, resorting to reproductive treatments abroad may bring some troubles when trying to register the child in patient's home country.^38^ Another interesting point to reflect on is that ROPA implies a new perspective on the biological dimension of motherhood because it distinguishes the gestational and genetic dimensions.^32^

### Motherhood

In the past, lesbians were only mothers in conjunction by step adopting the partner's child. This is the same as saying that in most cases, the maternity project was not a common project, often resulting from prior heterosexual relationships or as single women. Currently, more and more female couples are adopting or undergoing assisted reproduction as a common project *ad initium*.^39^

The impact of biological motherhood on the connection between the mother and the child is far from being clear. Some women believe the biological links affirm the social bond and the “role of mother”—it is not a “child of mine,” a “child of her,” it is a “child of hers.”^10^ On the other hand, some authors argue that there is no additional value attributable to ROPA by the mere distribution of biological ties. Furthermore, some argue that there is no intrinsic value in parents' biological ties to their children, and the distribution of roles within a parental project should be independent of biological considerations.^40^ However, the desire for partnership equality within lesbian couples is well established. Studies show that the absence of biological connection does not go unnoticed by these couples. Although lesbians show more equity in childcare than heterosexuals, birth mothers have this more marked than their partners—studies show that children born after single parented methods search for the biological mother when they are hungry and the other mother when they want to play.^41^

In general, women prefer to have an active and equal role in procreation and not being a mere spectator. It has been described as a way to eternalize the meeting of two people.^22^

Another interesting fact mentioned by some women is that in the case where one of them has a fertility problem and does not participate in the process, she often experiences mourning for her own fertility. In addition, nonbiological mothers often experience feelings of exclusion and jealousy.^41^

Nevertheless, some authors argue that the imperative of both women to participate actively and biologically in the procreation process may reflect a process of approximation to the heteronormative reality, thus reinforcing the feminine subordinate parenting. In addition, donor mothers often report feeling they are playing the role of “father.”^39^

In societies that do not accept LGBT motherhood, it can be very difficult to define the legal mother. The ROPA method can make this decision even more difficult because both are biological mothers, and therefore, under the law, both may be candidates. Similarly, from a social point of view, the process of acceptance by the social circle, including family, may also be more difficult because it is a more complex and somehow more unnatural process.^39^

Regarding the well-being of a child raised in a lesbian family, the evidence about the development of these children is reassuring. When compared with heteronormative families, children raised by 2 mothers show equal levels of self-esteem; school performance; social interaction; behavioral, psychological and cognitive development; sexual orientation; and gender identification. In fact, the criteria for adoption are stricter than those for assisted reproduction, so one may assume that couples able to adopt must also be able to have a child by ART.^26,42,43^

### Quantitative research

Until date, 8 studies have been published describing the outcomes of the ROPA method, 6 case series and 2 cohort studies (Table I).

**Table 1:** Outcomes of ROPA cycles reported by the 8 studies of quantitative research

Rates	Marina et al*	Royo et al	Yeshua et al	Bodri et al	Nuñez et al†	Brandão et al	Brandão et al	Diego et al
Rates per embryo transfer								
Positive pregnancy test rate	—	—	—	60%	70.0% (IVF: 47.5%; *P* = .004)	—	63.3% (IVF: 58.3%; *P*=.27)	—
Clinical pregnancy rate	46%	—	—	52%	60.0% (IVF: 40.0%; *P* = .011)	—	57.0% (IVF: 50.2%; *P*=.15)	—
Miscarriage rate	15%	—	—	—	—	—	17.2% (IVF: 16.9%; *P*>.99)	—
Live-birth rate	30.8%‡	—	—	41.9%	57.1% (IVF: 29.8%; *P* = .001)	—	46.1% (IVF: 40.9%; *P*=.14)	—
Rates per ROPA cycle								
Positive pregnancy test rate	—	58%	69.4%	—	81.7% (IVF: 64.2%; *P* = .016)	—	—	—
Clinical pregnancy rate	—	42%	—	—	71.7% (IVF: 55.8%; *P* = .04)	—	—	77% (IVF: 50%)
Miscarriage rate	—	15%	—	—	—	—	—	7% (IVF: 6%)
Live-birth rate	—	—	25%	60%	66.1% (IVF: 43.4%; *P* = .005)	79%	73.7%	61% (IVF: 42%)
Rate per pregnancy								
Multiple pregnancy rate	7.7%	—	—	14%	—	4.5%	—	—

* Only includes fresh embryo transfers.

† Percentages for ROPA compared with single-way IVF.

‡ Potential LBR because it includes 3 ongoing pregnancies.

The first case series was published in 2010 reporting 14 cycles in 14 couples. The mean age of the donor patients was 35.1 years and of the recipients was 34.6 years. The mean number of mature oocytes (MII) and embryos obtained per cycle was 9.4 and 5, respectively. Thirty five percent of the couples had surplus embryos to freeze, but 1 couple had no viable embryos. The authors only report the outcomes of the fresh embryo transfer. The clinical pregnancy rate (CPR) was 46%. They reported 1 live newborn and 3 ongoing pregnancies at the time of the study, so there was a 15% of miscarriage rate (MR) and a 30.8% of potential LBR. Most of the cases were double embryo transfer resulting in 1 (7.7%) twin pregnancy.^44^

In 2014, another Spanish group published a case series of lesbian couples undergoing ART. They had 8 cycles of ROPA with a positive pregnancy test rate of 58%, 42% of CPR, and 15% of MR per cycle, which they considered similar to those of conventional IVF.^45^

In 2015, a series of 20 couples and 36 ROPA cycles was published. The mean age of the donor patients was 35.7 years and of the recipients was 38.1 years. The rate of positive pregnancy test was 69.4% per cycle and 66.7% per couple (including all cycles). In the end, the LBR was 25% per cycle and 42.9% per couple.^46^

The largest case series was published in 2018 based on 141 ROPA cycles performed by 121 couples. Statistically significant differences were found between donors' and recipients' basal characteristics—recipients had more previous births and previous ART, as opposed to donors who had better ovarian reserve. No differences were found concerning age or BMI. Forty percent of the cycles were due to medical indications: failed previous inseminations, advanced female age, or low ovarian reserve. The cumulative LBR (rate per couple) was 60%, with a twin rate of 14%, prematurity rate of 17.7% (mainly due to multiple pregnancies), and cesarean section rate of 47.2%. Most of the cycles (88%) were synchronous, meaning they ended in a fresh embryo transfer. Nevertheless, no differences in the outcomes were found between this and the freeze-all group.^1^

In 2022, a study describing the pathway of female couples in a fertility clinic included 129 couples and found a higher LBR per couple in ROPA compared with that of single-sided IVF (79 versus 58%). The authors hypothesize this could be explained by the fact that ROPA offers the possibility of choosing the best of each side. Nevertheless, it is important to bear in mind that this was based in only 25 ROPA cycles.^23^

Other study from 2022 reported 31 cycles of ROPA. They found a CPR of 77%, LBR of 7%, and MR of 7%. All these rates were higher compared with one-way IVF, but the authors did no statistical comparison.^47^

The first comparative study published was a retrospective cohort based on 60 couples (and cycles) of ROPA and 120 cycles of IVF. No differences were found in the age of the ROPA donors, recipients, and women undergoing single parented IVF (around 34 years old), but the partners of the latter were significantly older (36.5 years old, *P*=.001). On the other hand, the antral follicle count was significantly higher in the ROPA donors compared with the IVF patients (17.4 and 14.6, respectively, *P*=.045). The number of mature oocytes retrieved was significantly higher with ROPA (9.4 vs. 7.8; *P*=.019), but there were no significant differences regarding the fertilization rate and the mean number of embryos obtained. Most of the embryo transfers (both groups) were double embryo transfer (81.7%) and in cleavage stage (87.2%). All rates of clinical outcomes analyzed were significantly better in the ROPA group, including positive pregnancy test, OPR, and live birth. The LBR per transfer was 57.1% and 29.8% for ROPA and IVF, respectively (*P*=.001), and the LBR per cycle (after all embryo transfers) was 66% for ROPA and 43.4% for IVF (*P*=.005). All these differences were also observed when multivariate analysis considering age, BMI, and number of MII was performed.^48^

The most recent quantitative study was also a cohort study comparing young patients without fertility disorders undergoing single-way IVF and ROPA. They included 99 ROPA cycles (73 couples), and 2929 IVF cycles with autologous oocytes (2334 patients) were included. There were no significant differences between donors and recipients regarding age, BMI, or AFC but comparing the ROPA and the non-ROPA samples, the mean age of the patients was 2.5 years higher in the non-ROPA group. The ROPA group obtained more mature oocytes (10.1 vs. 7.7; *P* < .01) and good quality embryos, according to the Spanish ASEBIR classification (embryos grade A: 0.59 vs. 0.44; *P* = .03; embryos grade B: 1.47 vs. 0.81; *P* < .01).

No significant differences were found in any of the clinical outcomes, including the rates of positive pregnancy test (63.3% vs. 58.3%; *P* = .27), clinical pregnancy (57% vs. 50.2%; *P* = .15), miscarriage (17.2% vs. 16.9%; *P* > .99), ectopic pregnancy (0% vs. 0.5%; *P* > .99), and live birth (46.1% vs. 40.9%; *P* = .14). Gestational age at delivery (39.1 weeks vs. 38.7 weeks; *P* = .17), preterm birth rate (7.9% vs. 12.1%; *P* = .61), and newborn weight (2809g vs. 3072g; *P* = .17) also had no significant differences. In the end, cumulative live-birth rate per cycle was 73.7% and 78.3% of the couples achieved at least 1 live birth in the course of all their ROPA cycles.^49^

## Discussion

With this study, we observed that there is a paucity of information regarding the ROPA method in scientific literature, despite its extensive use in some countries during the last decade.

ROPA allows both women of a lesbian couple to simultaneously be a biological mother of their child. Most of the women choose this method for this purpose, but others have a medical indication because one may choose the patient with the best ovarian and uterine prognosis for donor and recipient, respectively.^23^

As for any type of ART, ROPA has many ethical issues far from being universally accepted. These dilemmas are mainly related to the risks of a nonessential procedure and the well-being of the offspring raised by a same-sex couple. The risks of the procedure are similar to those of a conventional IVF, which is many times used in heterosexual couples who could also resort to a lower complexity treatment. Similarly, more and more studies report that children raised by same-sex couples have similar development compared with heteronormative families. However, most of the data available regarding this aspect are based on studies about adoption or AI. Studies addressing this double parented method are sparse and mainly based on the opinion of experts or women who have never undergone this treatment. It is still unclear the importance of sharing biological motherhood to mother-child bond, intracouple relationship, and self-fulfillment.^50^

National legislations vary considerably worldwide, not only regarding assisted reproduction or LGBT rights separately but also specifically the LGBT rights in the area of assisted reproduction. In most countries where ROPA is performed, there is no specific legislation. Thus, this method is assumed to be permitted just as any other legal reproductive treatment.^36,51^

There are only 8 studies addressing the clinical outcomes of this technique. All of them are retrospective studies, 6 cases series and 2 cohort study comparing ROPA with conventional one-way IVF. The largest case series included 141 cycles (120 couples), and the largest cohort study compared 99 and 2929 ROPA and single-way IVF cycles, respectively.^1,49^ Baseline characteristics of both donors and recipients were similar or presented minor differences in all studies. All studies report encouraging results. The LBR for fresh embryo transfer ranged between 25% and 57% and the total LBR per couple (including frozen embryo transfers) ranged from 31% to 79%. These are very good LBR for an IVF treatment, especially considering that most groups reported transfers of embryos in cleavage stage, which has lower rates of pregnancy compared with blastocyst. It is important to notice, though, that all the studies are based on small samples and 6 of them are case series.

Both comparative studies were retrospective cohorts comparing ROPA with conventional single-way IVF. Despite the small sample sizes, the authors were able to find similar or slightly better clinical outcomes with ROPA. This may be justified by the fact that with this method, one may choose the woman with best ovarian prognosis to be the donor and the woman with the best uterine status to be the recipient. By optimizing both oocyte quality and uterine environment, better outcomes may be expected.^49^

Being a narrative review, it was possible to make a more general and comprehensive approach to the subject, but the inclusion of all the information and the reproducibility of the work is not guaranteed. One of these limitations was the exclusion of articles by language, which in practice seemed to have had no major implications because these were only 2 papers without new data.

## Conclusion

The ROPA method is an alternative to conventional ART for lesbian couples that allows both women to share biological motherhood. It may be used simply for that purpose or due to medical indications choosing the woman with best ovarian reserve to be the donor and the woman with the best uterine status to be the recipient.

Although many couples seek for this kind of treatment, its role in promoting mother-child connection is still not clear, especially because most studies of lesbian motherhood are based on adoption or single parented reproductive treatments. Similarly, the ROPA method brings important ethical issues that are far from being universally accepted.

Despite having more than a decade of use, the outcomes of this technique have only been described in 8 retrospective studies, based on small samples. Nevertheless, studies report encouraging live-birth rates.

## Conflict of interest

The authors declare no conflicts of interest.
